# A comparison of structure, bonding and non-covalent interactions of aryl halide and diarylhalonium halogen-bond donors

**DOI:** 10.3762/bjoc.20.125

**Published:** 2024-06-27

**Authors:** Nicole Javaly, Theresa M McCormick, David R Stuart

**Affiliations:** 1 Department of Chemistry, Portland State University, 1719 SW 10th Ave, Portland OR 97201, United States

**Keywords:** aryl halide, diarylhalonium, halogen, halogen bond, non-covalent interaction

## Abstract

Halogen bonding permeates many areas of chemistry. A wide range of halogen-bond donors including neutral, cationic, monovalent, and hypervalent have been developed and studied. In this work we used density functional theory (DFT), natural bond orbital (NBO) theory, and quantum theory of atoms in molecules (QTAIM) to analyze aryl halogen-bond donors that are neutral, cationic, monovalent and hypervalent and in each series we include the halogens Cl, Br, I, and At. Within this diverse set of halogen-bond donors, we have found trends that relate halogen bond length with the van der Waals radii of the halogen and the non-covalent or partial covalency of the halogen bond. We have also developed a model to calculate Δ*G* of halogen-bond formation by the linear combination of the % p-orbital character on the halogen and energy of the σ-hole on the halogen-bond donor.

## Introduction

**Scheme 1:**
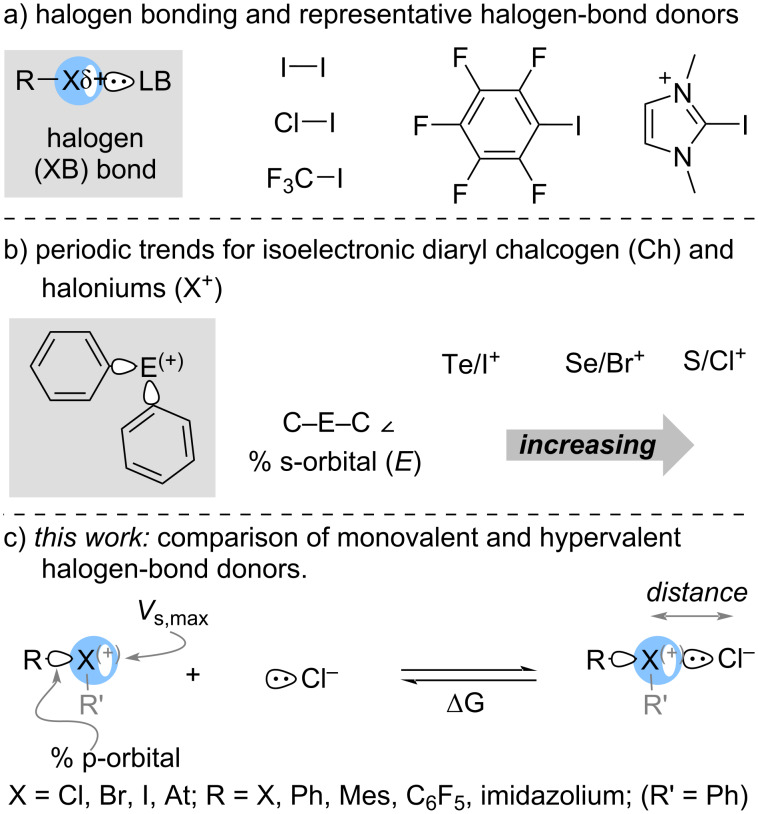
Overview of halogen bonding.

Halogen bonding has emerged as an important attractive interaction in a wide range of applications that include crystal engineering, drug discovery and light-emitting materials [1–4]. Although, halogen bonding was first “observed” over 200 years ago [5–6] and the structural characteristics were elucidated in the latter half of the nineteenth century [7], the term “halogen bond” entered the chemical literature in the latter half of twentieth century [8]. Detailed studies of halogen bonding that followed in the late 1990s and early 2000s primarily focused on inorganic molecular and interhalogens, and inorganic and organic halides that are monovalent (Scheme 1a) [1–4]. Hypervalent halogen compounds, specifically diaryliodonium salts, have also been known to form Lewis acid–base adducts [9–10] and a relative scale to quantify this property has recently been reported [11–12]. Consequently, there has been a recent surge in the use of diarylhalonium salts in halogen-bonding catalysis [13–19].

Crabtree has outlined the similarity in molecular orbitals (MO) formed in halogen bonds and hypervalent bonds (and hydrogen bonds) [20]. Recently, we [21], and Legault and Huber [22], independently investigated the connection between electronic structure (bonding) and molecular structure (geometry) in diarylhalonium salts. We found a periodic trend with respect to the percentage of s- and p-orbital character used by the central atom to bond to the aryl substituents for a series of isoelectronic diaryl chalcogen and diarylhalonium compounds (Scheme 1b) [21]. The amount of s-character in the orbital used by the central atom (both chalcogen and halogen) to bond with the aryl groups decreases moving down the respective group (16 and 17) [21]. We also found with a limited set of six compounds that the association constant (*K*_a_) for the halogen-bond interaction of diarylhalonium salts with pyridine decreased with increasing s-character used by the central halogen atom in the bond opposite the halogen bond; this is effectively the s-character in the σ*-orbital [21].

Conceptually, halogen-bond donors are commonly described by the electropositive σ-hole region, which is quantitatively described by *V*_s,max_ on the halogen, though other factors have also been considered (Scheme 1a) [1–4,23–25]. Huber and co-workers have posed the question: “Is There a Single Ideal Parameter for Halogen-Bonding Based Lewis Acidity?”, and concluded that, for a set of monovalent iodine-based halogen-bond donors, a linear combination of σ-hole and σ* energy provides a superior predictive ability than σ-hole alone [26]. In this work we compare a set of both monovalent and nominally hypervalent halogen-bond donors in which the central halogen atom is Cl, Br, I, and At. We have used density functional theory (DFT) to uncover periodic trends in the orbitals used by the central halogen atom in forming covalent and non-covalent interactions and how this impacts the interatomic distance and energy of halogen-bond interactions (Scheme 1c).

## Results and Discussion

**Scheme 2:**
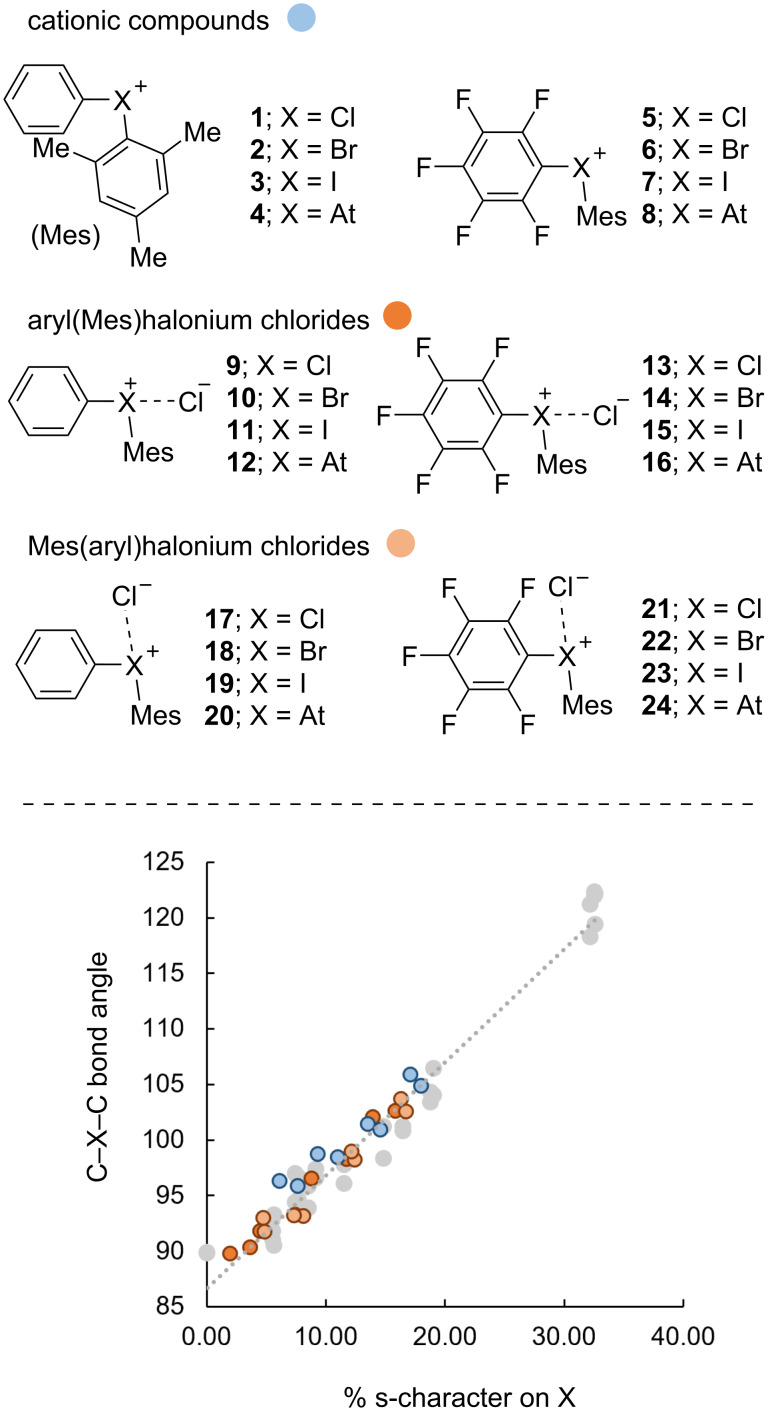
Correlation of orbital character on halogen with C–X–C bond angle for compounds **1**–**24**; data from prior work is represented by grey dots [21].

This study evolved from a parallel exploration of reactions involving unsymmetrical phenyl(mesityl)halonium salts, i.e., Ph(Mes)X^+^. DFT analysis revealed similar structural trends to our previous work [21] when we expanded the halogens to include astatine (At). Due to its radioactivity and short half-life it would be very challenging to synthesize astatine analogs of diarylhalonium salts and almost no experimental data exists on halogen bonding with astatine for comparison with DFT-generated data. However, the inclusion of molecules containing At in this study provides an opportunity to expand the theoretical framework describing the structure, bonding, and reactivity of diarylhalonium compounds [27]. Although some relativistic effects of astatine may not be sufficiently incorporated in calculations [28], others have shown in theoretical and limited experimental studies that astatine does engage in halogen-bonding interactions [29–30]. In this work, a series of halogen-bond donor molecules and their halogen bond complexes with chloride anion were optimized at the M062x/6-311+G(d) level of theory [31] with def2-tzvpp used for iodine and astatine, and with SMD solvation in tetrahydrofuran (THF) incorporating Huber, Truhlar, and Cramer’s correction for bromine and iodine [32] using Gaussian 09 [33]. Our prior work on the orbital analysis of diarylhalonium salts [21], showed good agreement between crystal structure data and energy-minimized structures at the B3LYP/cc-pvtz level with def2-qzvpp for iodine and tellurium and in the gas phase. In our present work using M06-2x/6-311+G(d) with def2-txvpp for iodine and astatine, we observed excellent agreement in the correlation between orbitals used on the halonium center and the C–X–C bond angle, i.e., molecular geometry, from our prior work (Scheme 2).

**Scheme 3:**
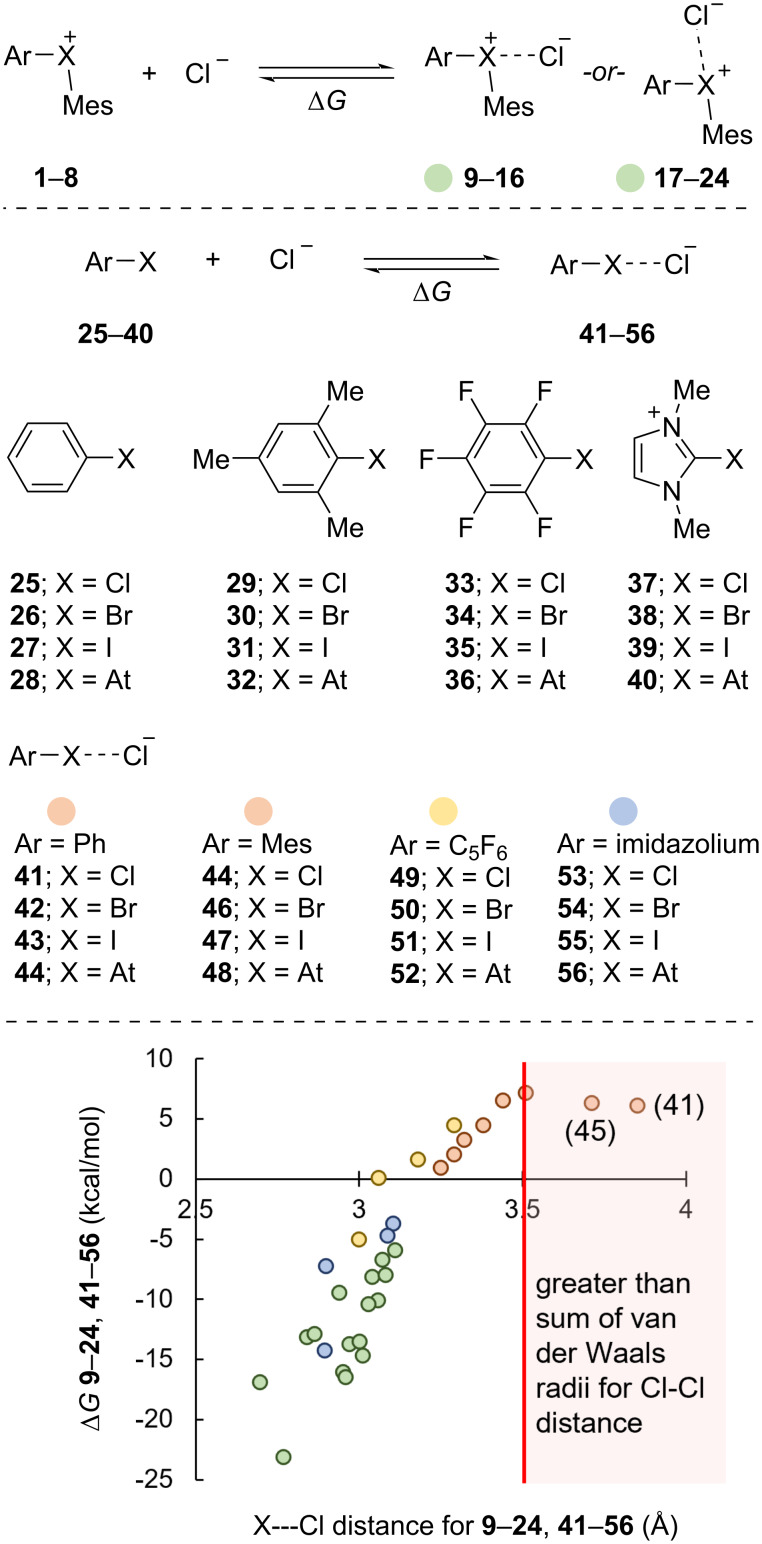
Correlation of Δ*G* for XB bond formation and X---Cl distance for compounds **9**–**24** (green dots), **41**–**48** (orange dots), **49**–**52** (yellow dots), and **53**–**56** (blue dots).

Given the similarities drawn between hypervalent and halogen bonding [20], we considered the association of the diarylhalonium cations **1**–**8** with chloride anion as well as the association of the monovalent subunits **25**–**36** with chloride anion (Scheme 3). We also considered the association of cationic monovalent halogen-bond donors **37**–**40** with chloride as the imidazolium iodide is a well-established core of halogen-bonding catalysts [34–35] (Scheme 3). In general, we observed that more exergonic association of the halogen-bond donors with chloride were associated with closer X---Cl contacts (Scheme 3). The monovalent halogen-bond donors of phenyl, mesityl, and pentafluorophenyl derivatives **25**–**35** had endergonic association with chloride (Scheme 3). Pentafluorophenyl astitide (**36**) was the only neutral monovalent halogen-bond donor with an exergonic association with chloride (Δ*G* = −5.0 kcal/mol). The X---Cl distance calculated for the halogen-bonding complexes **41** and **45** of phenyl chloride (**25**) and mesityl chloride (**29**) with chloride anion were 3.85 and 3.71 Å, respectively. These values are larger than the sum of the van der Waals radii (3.5 Å) for two chlorine atoms [36] and therefore unlikely to represent a substantial halogen-bonding interaction. The hypervalent halogen-bond donors **1**–**8** had substantially more exergonic association with chloride than their monovalent subunits. For instance, the association of chloride with pentafluorophenyl bromide (**34)** was Δ*G* = 1.6 kcal/mol, whereas the association of chloride with pentafluorophenyl(mesityl)bromonium (**6**) was Δ*G* = −13.2 kcal/mol. The overall charge on the halogen-bond donor also has an impact on the energy of association. The association of chloride with the diarylhalonium cations **1**–**8** had Δ*G* values that ranged from −5.9 to −23.1 kcal/mol (see Supporting Information File 1 for exact values). Likewise, the association of chloride with imidazolium halides **37**–**40** ranged from −3.7 to −14.3 kcal/mol, which overlaps with the range observed for the diarylhalonium cations.

**Scheme 4:**
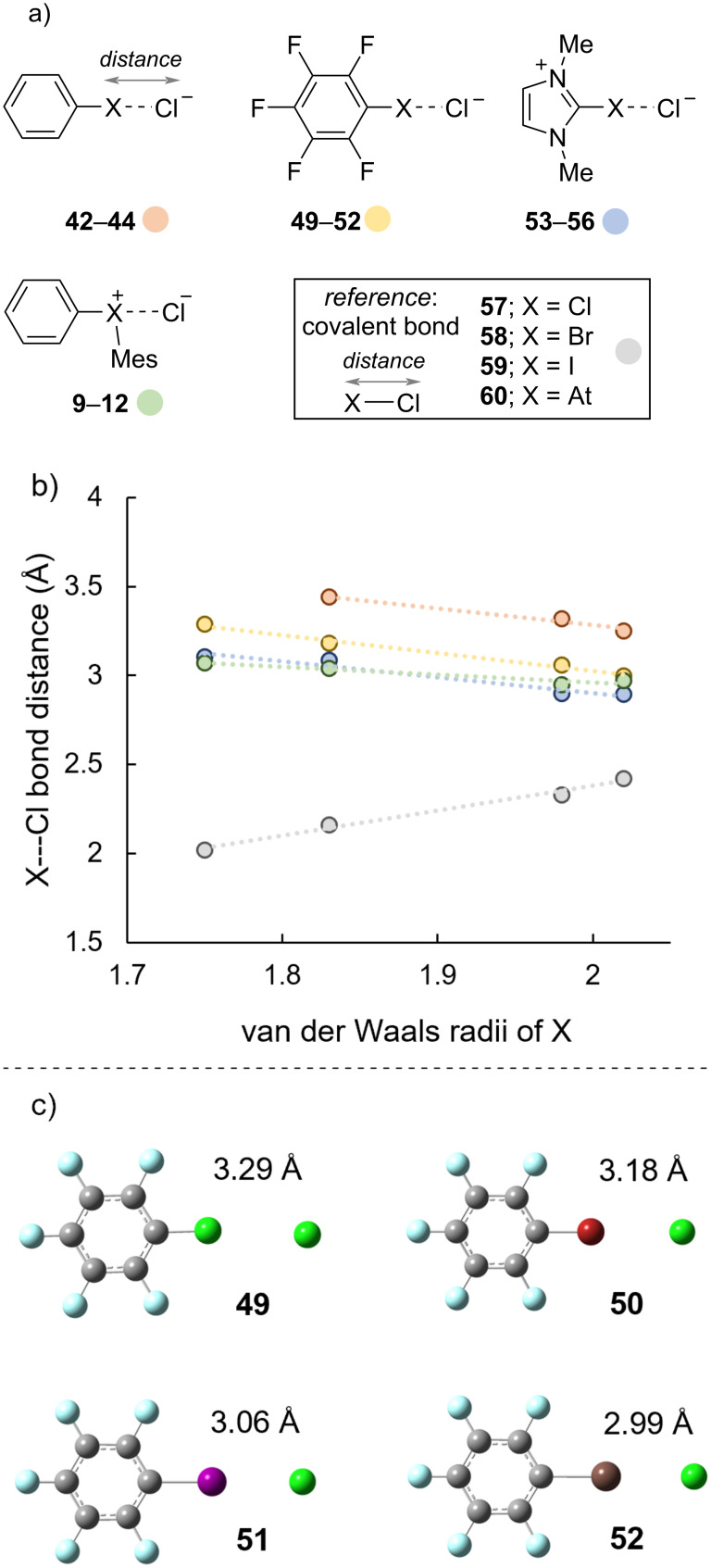
Correlation of X---Cl distance with van der Waals radii of X.

We delved deeper into the periodic trends related to the X---Cl distance for halogen-bond complexes **9**–**24**, **42**–**44**, and **46**–**56**; representative examples are shown in Scheme 4. As a reference we considered the trend in X–Cl covalent bond distance with respect to the van der Waals radii of X [36], and we observed a linear trend with a positive slope (Scheme 4b, grey dots). That is the length of the X–Cl (**57**–**60**) covalent bond increases with increasing van der Waals radii of X. Notably, this trend is also replicated for ionic bonds of the halides with sodium; longer ionic bond lengths are observed for larger halides [37]. On the other hand, halogen-bond complexes that we studied here revealed an opposite trend (Scheme 4b, orange, yellow, blue, and green dots). The halogen-bond length decreased with increasing van der Waals radii of X and the trend was more pronounced for monovalent halogen-bond donors. This is exemplified by halogen-bond complexes of the pentafluorophenyl halide series with chloride anion (Scheme 4c, **49**–**52**). A similar trend for decreasing bond length with increasing van der Waals radii has also been observed for some [38], though not all [39], series of chalcogen bonds. Generally, shorter bonds are stronger and longer bonds are weaker, and the trend we observe here for halogen bonding aligns with that rule of thumb (Scheme 3). The conceptual frameworks underpinning covalent and ionic bonds are orbital overlap and electrostatic attraction, respectively. Therefore, if larger van der Waals radii are associated with longer, weaker bonds where both these phenomena (orbital overlap and electrostatics) are operative (covalent and ionic bonds), our observations suggest a unique feature of halogen bonding that relates to bond length. Pauli repulsion and dispersion are additional factors that have been included in defining halogen bonds [25]. Smaller halogens that are less able to disperse lone-pairs may have greater destabilizing repulsive forces associated with them that ultimately lengthen the halogen bond relative to those of larger halogens [22,40].

**Scheme 5:**
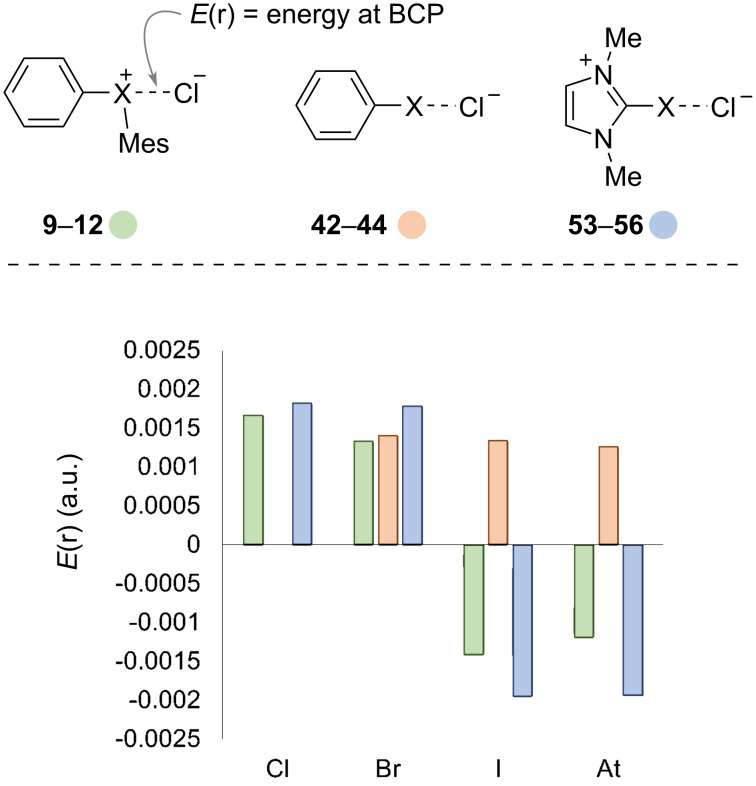
Comparison of *E*(r) for XB complexes **9**–**12**, **42**–**44**, and **53**–**56**.

Further analysis of the XB complexes revealed additional distinctions in the nature of the halogen bonds (Scheme 5). We used Bader’s quantum theory of atoms in molecules (QTAIM) [41] and assessed ρ(r), ∇2(r), and associated values. However, to minimize complexity we elected to focus on the distance between the bond critical points (BCP) and the atomic centers (available in Supporting Information File 1, Table S8) and the electronic energy at the BCPs, *E*(r) (Scheme 5). On the bonding continuum positive values of *E*(r) are generally associated non-covalent bonds and negative values of *E*(r) indicate increasing covalency [42]. We observed a switch from positive *E*(r) values for the lighter hypervalent phenyl(mesityl)haloniums **9** and **10** (X = Cl and Br) to negative *E*(r) values for the heavier haloniums **11** and **12** (X = I and At, Scheme 5, green bars). Uchiyama previously suggested that the diarylchloronium **9** has a “breakdown of the hypervalent bond” [43], and our data suggest that the interaction (halogen or hypervalent bond) between chloride anion and diarylchloronium cation **9** is non-covalent and likely dominated by electrostatic attraction. A similar switch from non-covalent halogen bond for the lighter (X = Cl and Br) to partially covalent halogen bond for the heavier (X = I and At) was also observed for the cationic imidazolium series of XB donors **53**–**56** (Scheme 5, blue bars). The neutral monovalent XB donors **26**–**28** formed halogen-bonding complexes **42**–**44** with non-covalent interactions in all cases, even those with heavier halogen iodine and astatine (Scheme 5, orange bars).

**Scheme 6:**
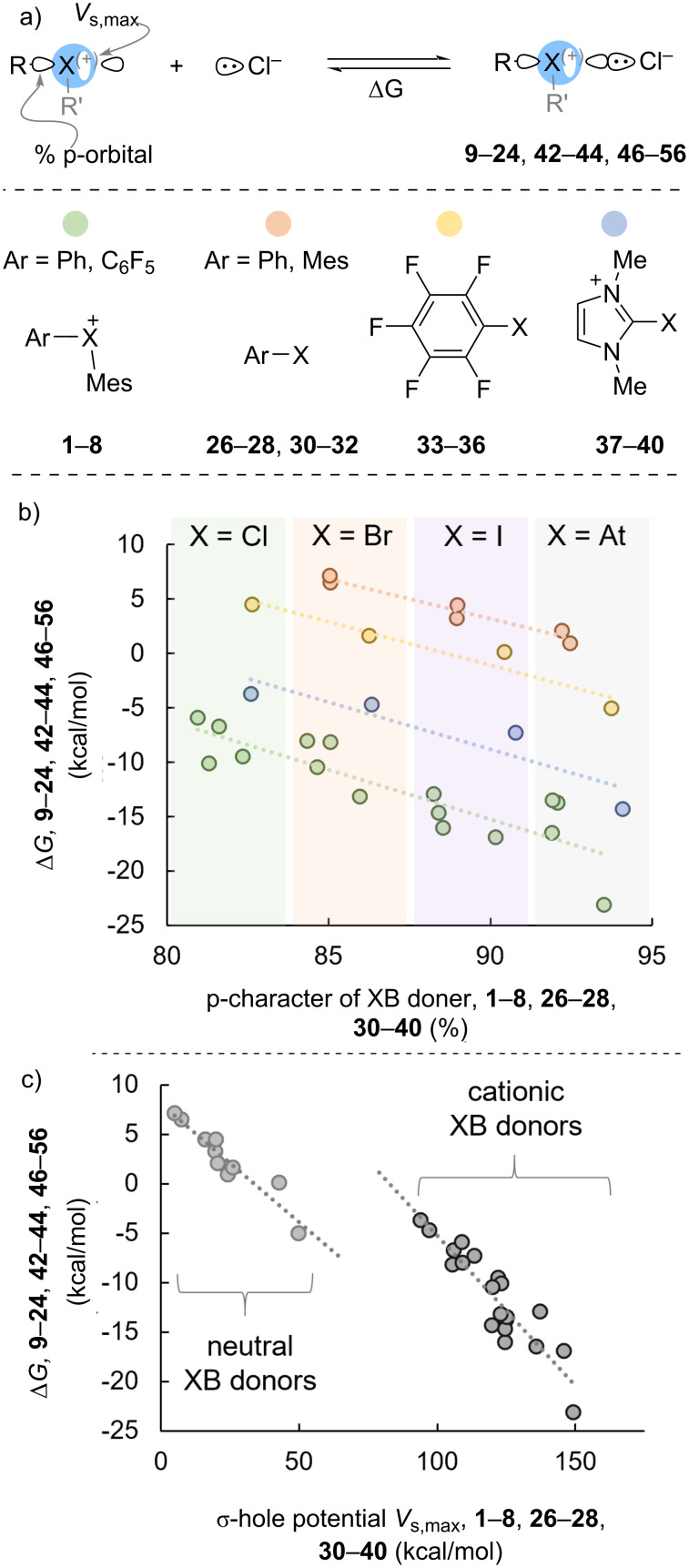
Correlation of p-character and *V*_s,max_ on X of XB donor with Δ*G* of XB bond.

We turned our attention from periodic trends in XB length to periodic trends in XB strength. The σ-bond oriented 180° relative to the halogen bond plays a central role in tuning the halogen bond properties [1–4]. Indeed, it impacts the size of the σ-hole (*V*_s,max_), and the energy of the σ* orbital has been shown be a key component of a predictive model for halogen-bond strength [26]. However, a confounding, though rarely discussed, factor for halogen-bond strength is the composition (s/p-character) of the orbital on the halogen atom that is engaged in the σ-bond (Scheme 6a). We have previously shown that larger association constants (*K*_eq_) were measured for hypervalent halogen-bond donors with greater calculated p-character on the halogen participating in the σ-bond opposite the hypervalent (or halogen) bond; both *K*_eq_ and p-character on X increased in the order Cl < Br < I [21]. We conducted a similar analysis here in which we plotted the percent p-orbital contribution on the XB donor against Δ*G* determined by DFT (Scheme 6b). Although, we found that this feature is a poor global predictor of Δ*G*, clear periodic trends are observed when related groups of XB donors are considered (Scheme 6b). When the halogen-bond donors are clustered into hypervalent **1**–**8** (Scheme 6b, green dots), monovalent aryl **26**–**28** and **30**–**32** (Scheme 6b, orange dots), perfluorophenyl **33**–**36** (Scheme 6b, yellow dots), and imidazolium **37**–**40** (Scheme 6b, blue dots) linear correlations with similar slopes are observed for p-orbital character and Δ*G* (Scheme 6b). Analysis in this way also provides an opportunity for comparison between these groups for the same halogen, that is a comparison between monovalent and hypervalent halogen-bond donors, and neutral and cationic halogen-bond donors. First, when the halogen is held constant it can be seen that the different classes of halogen-bond donors (monovalent vs hypervalent) use similar orbital composition to form the σ-bond with the aryl group (Scheme 6b). The percentage of p-character in the σ-orbital for halogen-bond donors with X = Cl is ≈80-82%, X = Br is ≈84–86%, X = I is ≈88–91%, and X = At is ≈92–94%. Interestingly, monovalent halogen-bond donors use slightly more p-orbital character to bond with the aryl group than their hypervalent counterparts. For example, in phenyl iodide (**27**) the iodine atom uses 88.97% p-character to bond with the phenyl group, whereas in phenyl(mesityl)iodonium cation (**3**) the iodine atom uses 88.54% p-character to bond with the phenyl group. Phenyl iodide (**27**) has three lone-pairs, whereas the phenyl(mesityl)iodonium cation (**3**) has two lone-pairs (though it does have another aryl group), and therefore this observation is consistent with Bent’s rule in which lone-pairs are stabilized by being in orbitals with more s-character [44]. An additional observation regarding the p-character directed at the aryl group by the halogen center relates to the charge on the halogen-bond donor. The halogen of the cationic imidazolium halogen-bond donors **37**–**40** has the largest amount of p-orbital character in bonding with the aryl group, this was followed by the perfluorophenyl monovalent halogen bond donors **33**–**36**, then monovalent aryl halogen-bond donors **26**–**28** and **30**–**32**, and finally hypervalent halogen-bond donors **1**–**8**. This observation is also consistent with Bent’s rule in which greater p-character is directed toward more electronegative ligands [44].

We also consider a correlation between *V*_s,max_ of the halogen-bond donor and Δ*G* of the halogen bond (Scheme 6c) [45]. Although a modest linear correlation (R^2^ = 0.90) was observed over all data points, in which XB donors with larger *V*_s,max_ values also had more exergonic associations, we actually observed two almost parallel clusters of data (Scheme 6c). In this case, neutral XB donors **26**–**28**, and **30**–**36** had *V*_s,max_ values ≈5–50 kcal/mol and a slope of −0.24 (Scheme 6c, light grey dots), whereas cationic XB donors **1**–**8** and **37**–**40** had *V*_s,max_ values ≈100–150 kcal/mol and a slope of −0.30 (Scheme 6c, dark grey dots). So, the distinction in our data set regarding *V*_s,max_ is not between monovalent and hypervalent halogen-bond donors, but rather between neutral and cationic halogen-bond donors. Three additional points regarding these data sets warrant comment. First, within the neutral XB donors it is perhaps not surprising that perfluoroaryl XB donors **32**–**36** had substantially larger *V*_s,max_ values than their non-fluorinated counter parts. Second, two data points are especially representative of the discontinuity in these data sets, the pentafluorophenyl astitide XB donor **36** has a *V*_s,max_ = 49.8 kcal/mol and Δ*G* = −5.0 kcal/mol, on the other hand cationic imidazolium chloride XB donor **37** has a *V*_s,max_ = 94.0 kcal/mol (almost two fold that of **36**) yet has a Δ*G* = −3.7 kcal/mol (less than that of **36**). Third, within the cationic XB donors the hypervalent haloniums **1**–**8** in which the positive charge is primarily located on the halogen had larger *V*_s,max_ values than the imidazolium halides **37**–**40** in which the positive charge is primarily delocalized on the imidazolium ring.

**Scheme 7:**
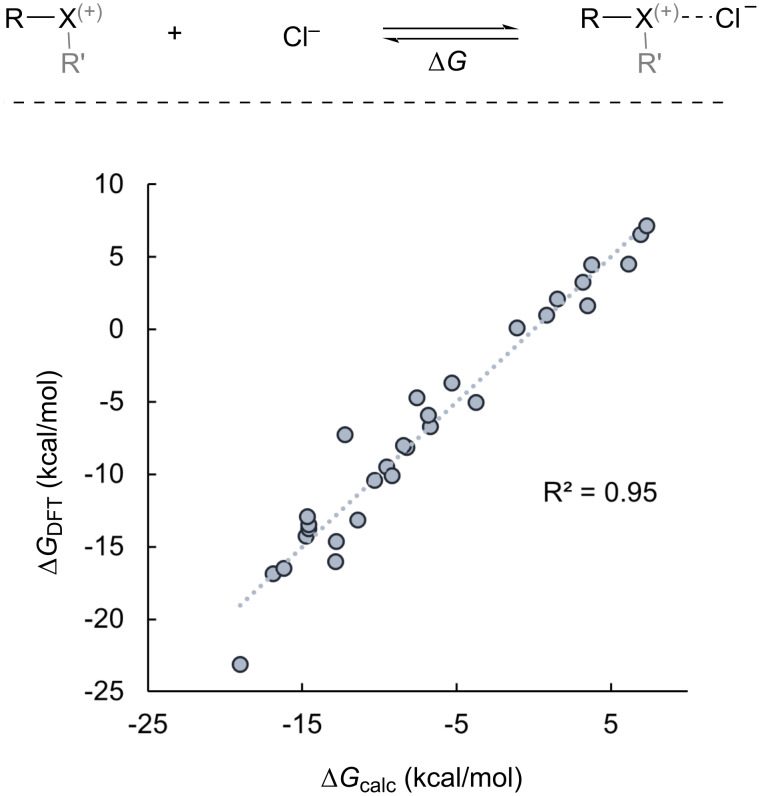
Model for Δ*G* based on Equation 1.

We have developed a model for Δ*G* of the halogen bonds investigated in this work by merging the two concepts of p-orbital character and *V*_s,max_ of the XB donor (Scheme 7). Although we considered other characteristics of XB donors, including NPA charges and Hirshfeld charges (available in Supporting Information File 1, Table S5), the linear combination of p-character (%) and σ-hole (*V*_s,max_) provided the highest correlation based on linear regression analysis of Δ*G*_DFT_ vs Δ*G*_calc_, wherein Δ*G*_calc_ is obtained from Equation 1 [46].


[1] $$\Delta G_{\text{calc}}=-1.95\text{ (\% p orbital})-7.56\text{ (}V_{\text{s,}\max})-6.78$$

Our model was developed with normalized parameters of % p-orbital character and *V*_s,max_ and therefore a comparison of the parameter coefficients reveals that *V*_s,max_ is a more dominant term than % p-orbital character in predicting Δ*G* (Equation 1 and Scheme 7). However, the % p-character term is non-negligible and demonstrates that this highly intuitive parameter contributes to the prediction of Δ*G* for halogen bonding. It is important to point out that this model is limited to the halogen-bond donors studied here and their interaction with chloride anion, although it is likely that prediction of Δ*G* with Equation 1 for structurally similar halogen-bond donors would be successful provided the parameters (% p-character and *V*_s,max_) are known. However, Δ*G* cannot be predicted for halogen-bond acceptors other than chloride and a more general predictive model should include parameters to describe the Lewis basic halogen-bond acceptor.

## Conclusion

In this work, we have compared the characteristics of monovalent, hypervalent, neutral, and cationic XB donors and their XB complexes with chloride anion by DFT. The structural characteristics of the diaryliodonium cations (XB donors) and diaryliodonium chloride salts (XB complexes) are consistent with our previous model that correlates s/p-orbital composition and C–X–C bond angle. The XB complexes that we studied generally follow the heuristic that stronger bonds are associated with shorter bonds. We found, however, that unlike covalent and ionic bonds, the halogen bonds studied decrease in length with increasing van der Waals radii of the halogen, and we suggest that this is possibly due to greater dispersive and lesser repulsive forces for larger halogens. This finding may prove useful in catalyst design where close spatial proximity of the substrate to other important structural information (i.e., chirality) has an impact on selectivity. Our analysis of selected XB complexes by QTAIM revealed that for cationic XB donors of the lighter halogens (X = Cl and Br) have non-covalent halogen bonds and those of the heavier halogens (X = I and At) have partially covalent halogen bonds. Clustered analysis of the XB donor parameters % p-orbital character and σ-hole potential (*V*_s,max_) showed linear correlations with Δ*G*_DFT_ of the halogen bond. The linear combination of the normalized parameters (% p-orbital character and *V*_s,max_) provides a model to calculate Δ*G* of the halogen bond.

## Supporting Information

File 1 Computational data.

File 2 Coordinates of optimized structures.

## Data Availability

All data that supports the findings of this study is available in the published article and/or the supporting information to this article.

## References

[1] Cavallo G, Metrangolo P, Milani R, Pilati T, Priimagi A, Resnati G, Terraneo G (2016). Chem Rev.

[2] Beale T M, Chudzinski M G, Sarwar M G, Taylor M S (2013). Chem Soc Rev.

[3] Gilday L C, Robinson S W, Barendt T A, Langton M J, Mullaney B R, Beer P D (2015). Chem Rev.

[4] Kolář M H, Hobza P (2016). Chem Rev.

[5] Colin M M, Gaultier de Claubry H (1814). Ann Chim (Cachan, Fr).

[6] Colin M M (1814). Ann Chim (Cachan, Fr).

[7] Guthrie F (1863). J Chem Soc.

[8] Zingaro R A, Hedges R M (1961). J Phys Chem.

[9] Ochiai M, Suefuji T, Miyamoto K, Tada N, Goto S, Shiro M, Sakamoto S, Yamaguchi K (2003). J Am Chem Soc.

[10] Ochiai M, Suefuji T, Shiro M, Yamaguchi K (2006). Heterocycles.

[11] Labattut A, Tremblay P-L, Moutounet O, Legault C Y (2017). J Org Chem.

[12] Mayer R J, Ofial A R, Mayr H, Legault C Y (2020). J Am Chem Soc.

[13] Zhang Y, Han J, Liu Z-J (2015). RSC Adv.

[14] Heinen F, Engelage E, Dreger A, Weiss R, Huber S M (2018). Angew Chem, Int Ed.

[15] Heinen F, Engelage E, Cramer C J, Huber S M (2020). J Am Chem Soc.

[16] Heinen F, Reinhard D L, Engelage E, Huber S M (2021). Angew Chem, Int Ed.

[17] Nishida Y, Suzuki T, Takagi Y, Amma E, Tajima R, Kuwano S, Arai T (2021). ChemPlusChem.

[18] Yoshida Y, Ishikawa S, Mino T, Sakamoto M (2021). Chem Commun.

[19] Yunusova S N, Novikov A S, Soldatova N S, Vovk M A, Bolotin D S (2021). RSC Adv.

[20] Crabtree R H (2017). Chem Soc Rev.

[21] Karandikar S S, Bhattacharjee A, Metze B E, Javaly N, Valente E J, McCormick T M, Stuart D R (2022). Chem Sci.

[22] Robidas R, Reinhard D L, Huber S M, Legault C Y (2023). ChemPhysChem.

[23] Řezáč J, de la Lande A (2017). Phys Chem Chem Phys.

[24] Riley K E, Hobza P (2013). Phys Chem Chem Phys.

[25] Thirman J, Engelage E, Huber S M, Head-Gordon M (2018). Phys Chem Chem Phys.

[26] Engelage E, Reinhard D, Huber S M (2020). Chem – Eur J.

[27] Rossi E, De Santis M, Sorbelli D, Storchi L, Belpassi L, Belanzoni P (2020). Phys Chem Chem Phys.

[28] Gamboni G, Belpassi L, Belanzoni P (2024). ChemPhysChem.

[29] Devore D P, Ellington T L, Shuford K L (2024). J Phys Chem A.

[30] Guo N, Maurice R, Teze D, Graton J, Champion J, Montavon G, Galland N (2018). Nat Chem.

[31] Wang Y, Verma P, Jin X, Truhlar D G, He X (2018). Proc Natl Acad Sci U S A.

[32] Engelage E, Schulz N, Heinen F, Huber S M, Truhlar D G, Cramer C J (2018). Chem – Eur J.

[33] (2013). Gaussian 09.

[34] Gliese J-P, Jungbauer S H, Huber S M (2017). Chem Commun.

[35] Sutar R L, Erochok N, Huber S M (2021). Org Biomol Chem.

[36] Mantina M, Chamberlin A C, Valero R, Cramer C J, Truhlar D G (2009). J Phys Chem A.

[37] (2024). CCCBDB bond length model; NIST Computational Chemistry Comparison and Benchmark Database; NIST Standard Reference Database Number 101, Release 22; May 2022; Russell, D. Johnson, III.

[38] Oliveira V, Cremer D, Kraka E (2017). J Phys Chem A.

[39] de Azevedo Santos L, van der Lubbe S C C, Hamlin T A, Ramalho T C, Matthias Bickelhaupt F (2021). ChemistryOpen.

[40] Ramasami P, Murray J S (2024). J Mol Model.

[41] Bader R F W (1991). Chem Rev.

[42] Miller D K, Chernyshov I Y, Torubaev Y V, Rosokha S V (2022). Phys Chem Chem Phys.

[43] Nakajima M, Miyamoto K, Hirano K, Uchiyama M (2019). J Am Chem Soc.

[44] Bent H A (1961). Chem Rev.

[45] Donald K J, Pham N, Ravichandran P (2023). J Phys Chem A.

[46] Santiago C B, Guo J-Y, Sigman M S (2018). Chem Sci.

