# Supplementary material for: A comparison of structure, bonding and non-covalent interactions of aryl halide and diarylhalonium halogen-bond donors
Source: Beilstein J Org Chem. 2024 Jun 27;20:1428–35. doi: 10.3762/bjoc.20.125 (PMC11216093; doi:10.3762/bjoc.20.125)
Supplement: File 1 — Computational data. [file Beilstein_J_Org_Chem-20-1428-s001.pdf]

BEILSTEIN JOURNAL OF ORGANIC CHEMISTRY

Supporting Information

for

A comparison of structure, bonding and non-covalent interactions of aryl halide and diarylhalonium halogen-bond donors

Nicole Javaly, Theresa M. McCormick and David R. Stuart

Beilstein J. Org. Chem. **2024,** *20,* 1428–1435. doi:10.3762/bjoc.20.125

Computational data

License and Terms: This is a supporting information file under the terms of the Creative Commons Attribution License (https://creativecommons.org/licenses/by/4.0). Please note that the reuse, redistribution and reproduction in particular requires that the author(s) and source are credited and that individual graphics may be subject to special legal provisions.
The license is subject to the *Beilstein Journal of Organic Chemistry* terms and conditions: (https://www.beilstein-journals.org/bjoc/terms)

Table of contents

Computational methods S2
Compounds included S3
Table S1: Compound numbers and structures, with associated halogens S3
Structural data S5
Table S2: Structural and energetic data for diarylhalonium compounds S5
Table S3: Structural and energetic data for aryl halides S6
Charge calculations S7
Table S4: Natural population, Hirshfeld, and Vs+ calculations for all compounds S7
NBO data S10
Table S5: Orbital contributions to bonds from hypervalent halogen centers S10
Table S6: Orbital contributions from X–Cl compounds S10
Table S7: Orbital contributions to bonds from monovalent halogen centers S11
QTAIM calculations S12
Table S8: Topology-based values at the X–Cl bond critical point (BCP). S12
References S13

Computational methods

All compounds were optimized at the M062x/6-311+G(d) level of theory with Def2-tzvpp used for Iodine and Astatine. Solvation in tetrahydrofuran (ε=7.4257) was included using the SMD solvation model with the altered van der waals radii for bromine and iodine defined by Huber *et al*[1].

M062x has been repeatedly shown to give good results for halogen bonding and hypervalent halogen compounds[2-6]. The combination of 6-311+G(d) and def2tzvpp was chosen to give a balance between accuracy for halogen bonding and computational efficiency[3, 7, 8].

Optimized structures were confirmed to contain no negative vibrational frequencies. All optimizations and frequency calculations were conducted using Gaussian09 with Gaussview6 for visualization[9].

Sigma hole potentials and Hirshfeld charges were calculated using Multiwfn, with the associated implementation of marching tetrahedra algorithm of Lu and Chen [10, 11] using the methodology described by Ravichandran *et al* [12]. Natural population analysis based charges and orbital contributions to bonds were calculated using NBO7.0[13, 14].

Compounds included

Table S1: Compound numbers and structures, with associated halogens

	Compound Code	halogen	name
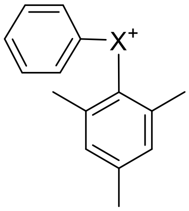	1	Cl	Ph(Mes)Cl+
	2	Br	Ph(Mes)Br+
	3	I	Ph(Mes)I+
	4	At	Ph(Mes)At+
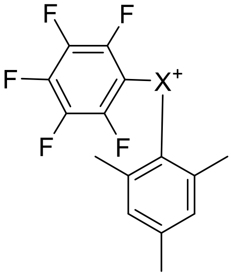	5	Cl	5FPh(Mes)Cl+
	6	Br	5FPh(Mes)Br+
	7	I	5FPh(Mes)I+
	8	At	5FPh(Mes)At+
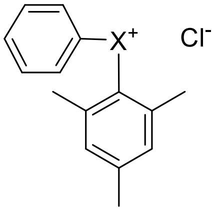	9	Cl	Ph(Mes)Cl+Cl-
	10	Br	Ph(Mes)Br+Cl-
	11	I	Ph(Mes)I+Cl-
	12	At	Ph(Mes)At+Cl-
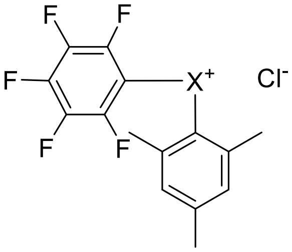	13	Cl	5FPh(Mes)Cl+Cl-
	14	Br	5FPh(Mes)Br+Cl-
	15	I	5FPh(Mes)I+Cl-
	16	At	5FPh(Mes)At+Cl-
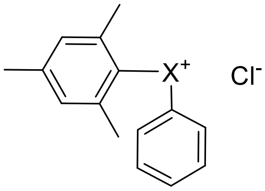	17	Cl	Mes(Ph)Cl+Cl-
	18	Br	Mes(Ph)Br+Cl-
	19	I	Mes(Ph)I+Cl-
	20	At	Mes(Ph)At+Cl-
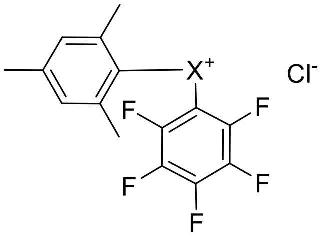	21	Cl	Mes(5Fph)Cl+Cl-
	22	Br	Mes(5FPh)Br+Cl-
	23	I	Mes(5FPh)I+Cl-
	24	At	Mes(5FPh)At+Cl-

	Compound #	Halogen	Name
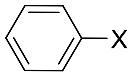	25	Cl	PhCl
	26	Br	PhBr
	27	I	PhI
	28	At	PhAt
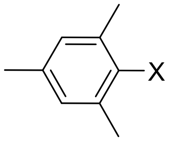	29	Cl	MesCl
	30	Br	MesBr
	31	I	MesI
	32	At	MesAt
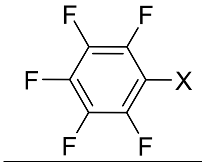	33	Cl	5fph-cl
	34	Br	5fph-br
	35	I	5fph-i
	36	At	5fph-at
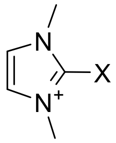	37	Cl	Cl-imidazole
	38	Br	Br-imidazole
	39	I	I-imidazole
	40	At	At-imidazole
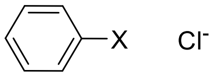	41	Cl	PhCl-Cl
	42	Br	PhBr-Cl
	43	I	PhI-Cl
	44	At	PhAt-Cl
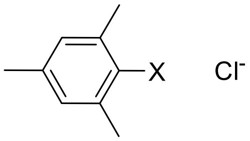	45	Cl	MesCl-Cl
	46	Br	MesBr-Cl
	47	I	MesI-Cl
	48	At	MesAt-Cl
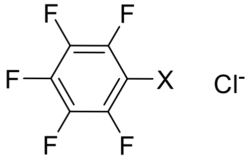	49	Cl	5fphcl-cl
	50	Br	5fphbr-cl
	51	I	5fphi-cl
	52	At	5fphat-cl
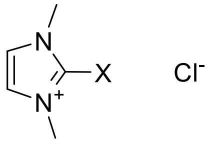	53	Cl	Cl-imidazole -Cl
	54	Br	Br-imidazole -Cl
	55	I	I-imidazole -Cl
	56	At	At-imidazole -Cl
X−Cl	57	Cl	Cl2
	58	Br	BrCl
	59	I	ICl
	60	At	AtCl

Structural data

Table S2: Structural and energetic data for diarylhalonium compounds.
All distances are reported in Ångstroms, angles in degrees, and energies in kcal/mol. Electronic energy with thermal free energy correction is reported as EE+TFE. Abbreviations are as follows: halogen is given as X, ipso carbon on phenyl rings as C(Ph), ipso carbon on mesityl ring as C(Mes), and chloride anion as Cl.

Molecule		Energy (kcal/mol)	Structure (Å, °)					
Code	halogen	Association ΔG	Halogen-Phenyl Carbon distance	Halogen-Mesityl carbon distance	X-Cl distance	C(Ph)-X-Cl angle	C(Mes)-X-Cl angle	C-X-C angle
19	I	-14.6592	2.11065	2.11759	3.00873	84.99325	178.1896	93.19639
11	I	-16.031	2.12269	2.10314	2.95431	175.3882	82.12929	93.27887
18	Br	-8.00199	1.95076	1.94284	3.07707	87.1195	174.6581	98.21514
10	Br	-8.14695	1.94731	1.94233	3.04411	177.6389	82.51125	98.27851
17	Cl	-5.90862	1.80985	1.79736	3.10504	88.01765	169.3155	102.598
9	Cl	-6.71811	1.8034	1.80234	3.0653	173.1908	83.32032	102.6054
20	At	-13.4996	2.19931	2.21885	3.00027	86.40401	178.1705	91.77632
12	At	-13.745	2.21966	2.19531	2.9697	175.7699	84.06511	91.82732
13	Cl	-9.47413	1.7649	1.83011	2.93915	169.6177	84.60099	102.0512
14	Br	-13.1476	1.93023	1.96058	2.83957	174.8097	85.38072	96.57637
15	I	-16.8769	2.15723	2.11083	2.69563	175.056	85.49545	90.33305
16	At	-23.1099	2.25606	2.20061	2.76898	175.9262	86.46664	89.82329
21	Cl	-10.0816	1.7504	1.8194	3.05773	76.97296	170.41	103.7151
22	Br	-10.4242	1.89196	1.95639	3.02762	77.94349	174.3047	99.00253
23	I	-12.9072	2.06975	2.13049	2.86311	79.80682	172.8553	93.22367
24	At	-16.4778	2.16091	2.22518	2.95731	80.06043	172.9478	93.0209
5	Cl		1.75288	1.83199				105.8797
6	Br		1.89586	1.96545				101.4374
7	I		2.07159	2.11398				98.76357
8	At		2.16407	2.20749				96.35432
3	I		2.10706	2.11079				98.44635
2	Br		1.9521	1.94365				100.9746
1	Cl		1.80985	1.80574				104.8866
4	At		2.19614	2.20157				95.8945

Table S3: Structural and energetic data for aryl halides. All distances are reported in Ångstroms, angles in degrees, and energies in kcal/mol. Electronic energy with thermal free energy correction is reported as EE+TFE.

Molecule		Energy (kcal/mol)	Structure (Å, °)		
code	halogen	Association ΔG	Halogen - Carbon distance	Halogen–Chloride distance	C-X-Cl angle
25	Cl		1.75283		
26	Br		1.90757		
27	I		2.09647		
28	At		2.18713		
29	Cl		1.75807		
30	Br		1.91601		
31	I		2.1075		
32	At		2.19993		
33	Cl		1.71821		
34	Br		1.87332		
35	I		2.06831		
36	At		2.16238		
37	Cl		1.68822		
38	Br		1.8446		
39	I		2.04559		
40	At		2.143		
41	Cl	6.08872	1.748	3.84731	174.8494
42	Br	6.526094	1.90585	3.44397	179.9923
43	I	3.264929	2.10344	3.32225	177.1983
44	At	0.963854	2.20263	3.24867	177.9025
45	Cl	6.299563	1.75356	3.70862	175.6576
46	Br	7.143562	1.91284	3.51423	179.9484
47	I	4.462844	2.11216	3.37545	177.0793
48	At	2.075172	2.21331	3.28858	177.8118
49	Cl	4.486689	1.71954	3.29347	175.971
50	Br	1635.519	1.87031	3.18421	179.8712
51	I	0.111697	2.10508	3.05746	178.1154
52	At	-5.02948	2.21955	2.99999	179.6705
53	Cl	-3.69352	1.69016	3.10458	176.4315
54	Br	-4.70632	1.85728	3.08581	179.9841
55	I	-7.26091	2.10126	2.89723	179.985
56	At	-14.2733	2.21914	2.89444	179.9606
57	Cl			2.02167	
58	Br			2.16046	
59	I			2.33182	
60	At			2.42567	

Charge calculations

Table S4: Natural population, Hirshfeld, and Vs+ calculations for all compounds.

Calculation methods for all values described above. Abbreviations are as follows: NPA is Natural Population Analysis Charge, pPh is for a sigma hole that is opposite a phenyl ring, pMes is for a sigma hole opposite a mesityl ring, X is for halogen, Mes and Ph refer to the ipso carbons on those rings, Cl- is for chloride anion, Cl#2 is for, in halogen dichloride molecules, the secondary chloride associated with the halogen.

Molecule		Natural Population Analysis Charges					Sigma Hole Potentials (kcal/mol)		Hirshfeld Charges				
code	name	NPA X	NPA Mes	NPA Ph	NPA Cl	NPA Cl2	pPh sigma hole kcal/	pMes sigma hole	Hirshfeld X	Hirshfeld Ph(X)	Hirshfeld Mes(X)	Hirshfeld Cl-	Hirshfeld Cl #2
25	PhCl	-0.018		-0.0502			1.85581		-0.0847	0.01426			
26	PhBr	0.04755		-0.1159			7.47988		-0.0668	-0.0058			
27	PhI	0.14862		-0.1957			19.6049		-0.0352	-0.0213			
28	PhAt	0.13922		-0.1761			24.2192		-0.0374	-0.0243			
57	Cl2	0			0		25.2123		0			0	
58	BrCl	0.09909			-0.0991		36.5404		0.04373			-0.0437	
59	ICl	0.26277			-0.2628		53.6514		0.10422			-0.1042	
60	AtCl	0.3402			-0.3402		72.8097		0.16248			-0.1625	
41	PhCl-Cl	0.006		-0.0557	-0.9975				-0.0922	0.01213		-0.9663	
42	PhBr-Cl	0.09449		-0.1321	-0.9855				-0.0808	-0.0145		-0.9255	
43	PhI-Cl	0.18382		-0.2233	-0.9637				-0.102	-0.0356		-0.867	
44	PhAt-Cl	0.19885		-0.2246	-0.9488				-0.1092	-0.0484		-0.8281	
45	MesCl-Cl	-0.0163		0.04862	-0.9963				-0.071		-0.0006	-0.9603	
46	MesBr-Cl	0.08237		-0.119	-0.9883				-0.052		-0.0249	-0.9324	
47	MesI-Cl	0.16862		-0.2254	-0.9688				-0.0739		-0.0457	-0.8776	
48	MesAt-Cl	0.17612		-0.1449	-0.9542				-0.0837		-0.0575	-0.8389	
29	MesCl	-0.0417		0.04732				-0.1435	-0.0646		0.00214		
30	MesBr	0.03458		-0.1198				5.0016	-0.0451		-0.0172		

31	MesI	0.12973		-0.197			16.061	-0.0186		-0.0331	
32	MesAt	0.11374		-0.0985			20.592	-0.0241		-0.0354	
19	Mes(Ph)I+Cl-	1.06067	-0.2153	-0.2061	-0.8974			0.46288	-0.0097	-0.0435	-0.7124
11	Ph(Mes)I+Cl-	1.06528	-0.2148	-0.2559	-0.88			0.45987	-0.0311	-0.0243	-0.6848
18	Mes(Ph)Br+Cl-	0.74745	-0.0857	-0.9414	-0.0936			0.36629	0.01287	-0.0195	-0.7937
10	Ph(Mes)Br+Cl-	0.74842	-0.0365	-0.1345	-0.9343			0.3681	-0.0047	-0.0021	-0.7749
17	Mes(Ph)Cl+Cl-	0.57155	0.00492	-0.02	-0.9672			0.31183	0.03477	0.00998	-0.8374
9	Ph(Mes)Cl+Cl-	0.57297	0.03954	-0.0452	-0.9617			0.31373	0.02739	0.01893	-0.8147
20	Mes(Ph)At+Cl-	1.07862	-0.2694	-0.184	-0.8884			0.46279	-0.0101	-0.0541	-0.683
12	Ph(Mes)At+Cl-	1.07885	-0.1897	-0.2593	-0.8793			0.46199	-0.0439	-0.0247	-0.671
3	Ph(Mes)I+					124.528	124.636	0.54643	-0.0039	-0.0217	
2	Ph(Mes)br+	0.68342	-0.056	-0.1011		105.476	109.075	0.38482	0.01172	-0.0054	
1	Ph(Mes)cl+	0.53031	0.02265	-0.0299		106.074	109.026	0.32785	0.03654	0.0175	
4	Ph(Mes)at+	0.98332	-0.1364	-0.1862		125.069	125.347	0.54017	-0.0065	-0.0222	
33	5fph-cl	0.07596		-0.1646		19.9627		-0.008	-0.0009		
34	5fph-br	0.15124		-0.2419		26.0063		0.01808	-0.0291		
35	5fph-i	0.27876		-0.323		42.8151		0.07448	-0.0468		
36	5fph-at	0.28128		-0.3138		49.7674		0.08127	-0.0541		
49	5fphcl-cl	0.10352		-0.1702	-0.9853			-0.0342	-0.0064		-0.9276
50	5fphbr-cl	0.19428		-0.2841	-0.9627			-0.0167	-0.0382		-0.8869
51	5fphi-cl	0.28651		-0.3537	-0.9137			-0.0394	-0.072		-0.787
52	5fphat-cl	0.30828		-0.3603	-0.8872			-0.0416	-0.0879		-0.7348
13	5FPh(Mes)Cl+Cl-	0.62019	-0.1865	0.05503	-0.9332			0.33602	0.0018	0.01783	-0.7703
14	5FPh(Mes)Br+Cl-	0.79879	-0.0798	-0.2854	-0.8587			0.38669	-0.0407	-0.0046	-0.6823
15	5FPh(Mes)I+Cl-	1.10664	-0.1644	-0.4057	-0.7441			0.46884	-0.0765	-0.0294	-0.5259
16	5FPh(Mes)At+Cl-	1.147	-0.193	-0.4153	-0.7674			0.4938	-0.0918	-0.0291	-0.5385
5	5FPh(Mes)Cl+	0.59101	0.03849	-0.1795		121.987	123.099	0.36427	0.01127	0.01889	
6	5FPh(Mes)Br+	0.75901	-0.0417	-0.2651		123.055	120.148	0.4312	-0.0199	-0.0047	
7	5FPh(Mes)I+	1.11375	-0.2134	-0.3587		145.793	137.344	0.61239	-0.037	-0.0206	
8	5FPh(Mes)At+	1.09898	-0.1355	-0.3458		149.278	135.854	0.61617	-0.0437	-0.0213	

21	Mes(5Fph)Cl+Cl-	0.6318	0.01305	-0.1513	-0.9557		0.34583	0.00779	0.00813	-0.8179
22	Mes(5FPh)Br+Cl-	0.82557	-0.0861	-0.2375	-0.9271		0.40976	-0.0194	-0.0235	-0.779
23	Mes(5FPh)I+Cl-	1.16065	-0.2736	-0.3519	-0.8466		0.50845	-0.0447	-0.0529	-0.6583
24	Mes(5FPh)At+Cl-	1.19095	-0.2305	0.37192	-0.872		0.53356	-0.0492	-0.0595	-0.6755
37	Climidazole	0.1337		0.41989		94.031	0.08323	0.14649		
38	Brimidazole	0.21013		0.34225		97.2126	0.11626	0.11146		
39	Iimidazole	0.34915		0.27117		113.55	0.19472	0.0948		
40	Atimidazole	0.34751		0.27857		119.837	0.19936	0.08584		
53	Climidazole -Cl	0.16057		0.41457	-0.9746		0.04408	0.13899		-0.9012
54	Brimidazole -Cl	0.24307		0.33013	-0.9494		0.06386	0.09835		-0.8562
55	Iimidazole -Cl	0.32157		0.24604	-0.8659		0.03485	0.06762		-0.7197
56	Atimidazole -Cl	0.34339		0.23203	-0.8478		0.03674	0.04716		-0.6795

NBO data

Table S5: Orbital contributions to bonds from hypervalent halogen centers

Orbital contributions are given as percentages.

Code	halogen	name	X-Mes S	X-Mes P	X-Mes D	X-Mes F	X-Ph S	X-Ph P	X-Ph D	X-Ph F
19	I	Mes(Ph)I+Cl-	8.12	91.4	0.42		11.3	88.4	0.43	
11	I	Ph(Mes)I+Cl-	12	87.6	0.35		7.43	92	0.54	
18	Br	Mes(Ph)Br+Cl-	12.4	87.1	0.47		14.5	85.1	0.45	
10	Br	Ph(Mes)Br+Cl-	15.3	84.3	0.41		11.7	87.4	0.53	
17	Cl	Mes(Ph)Cl+Cl-	16.7	82.8	0.48		17.7	81.9	0.45	
9	Cl	Ph(Mes)Cl+Cl-	18.7	80.9	0.43		15.8	83.7	0.52	
20	At	Mes(Ph)At+Cl-	4.83	94.8	0.24		7.8	91.8	0.22	
12	At	Ph(Mes)At+Cl-	8.25	91.5	0.17		4.47	95.1	0.32	
3	I	Ph(Mes)I+	11.2	88.4	0.29		11	88.5	0.38	
2	br	Ph(Mes)br+	15.3	84.3	0.34		14.6	85.1	0.4	
1	cl	Ph(Mes)cl+	18.7	81	0.39		18	81.6	0.42	
4	at	Ph(Mes)at+	7.81	91.9	0.15		7.61	92.1	0.2	
13	Cl	5FPh(Mes)Cl+Cl-	18.1	81.5	0.4		14	85.4	0.64	
14	Br	5FPh(Mes)Br+Cl-	15.1	84.5	0.41		8.79	90.6	0.63	
15	I	5FPh(Mes)I+Cl-	12.5	87	0.39	0.08	3.64	95.6	0.66	0.11
16	At	5FPh(Mes)At+Cl-	8.37	91.3	0.18	0.13	1.97	97.5	0.37	0.15
5	Cl	5FPh(Mes)Cl+	18.3	81.3	0.35		17.1	82.4	0.55	
6	Br	5FPh(Mes)Br+	15	84.6	0.33		13.5	86	0.5	
7	I	5FPh(Mes)I+	11.4	88.3	0.32	0.06	9.31	90.2	0.45	0.19
8	At	5FPh(Mes)At+	7.84	91.9	0.15	0.1	6.11	93.5	0.23	0.15
21	Cl	Mes(5Fph)Cl+Cl-	16.3	83.2	0.45		17.3	82.1	0.57	
22	Br	Mes(5FPh)Br+Cl-	12.2	87.4	0.46		13.9	85.6	0.56	
23	I	Mes(5FPh)I+Cl-	7.29	92.2	0.49	0.07	10.2	89.2	0.5	0.1
24	At	Mes(5FPh)At+Cl-	4.74	94.9	0.26	0.09	6.64	92.9	0.26	0.18

Table S6: Orbital contributions from X–Cl compounds

Orbital contributions are given as percentages.

code	halogen	name	X-Cl S	X-Cl P	X-Cl D	X-Cl F
57	Cl	Cl2	6.37	92.8	0.87	
58	Br	BrCl	5.11	93.8	1.1	
59	I	ICl	3.87	94.6	1.27	0.26
60	At	AtCl	2.11	96.4	1.08	

Table S7: Orbital contributions to bonds from monovalent halogen centers

Orbital contributions are given as percentages.

Molid	halogen	name	X-Mes S	X-Mes P	X-Mes D	X-Mes F	X-Ph S	X-Ph P	X-Ph D	X-Ph F
37	Cl	Climidazole					16.7	82.6	0.71	
38	Br	Brimidazole					13.1	86.3	0.61	
39	I	Iimidazole					8.6	90.8	0.5	0.11
40	At	Atimidazole					5.47	94.1	0.27	0.17
25	Cl	PhCl					18	81.5	0.53	
26	Br	PhBr					14.5	85.1	0.47	
27	I	PhI					10.6	89	0.39	0.08
28	At	PhAt					7.2	92.5	0.21	0.13
29	Cl	MesCl	18.7	80.8	0.54					
30	Br	MesBr	14.5	85	0.44					
31	I	MesI	10.6	89	0.33	0.07				
32	At	MesAt	7.47	92.2	0.18	0.13				
33	Cl	5fph-cl					16.7	82.6	0.62	
34	Br	5fph-br					13.2	86.3	0.55	
35	I	5fph-i					9	90.4	0.48	0.09
36	At	5fph-at					5.84	93.8	0.25	0.15
41	Cl	PhCl-Cl					35.6	82.1	0.57	
42	Br	PhBr-Cl					12.8	86.7	0.58	
43	I	PhI-Cl					8.62	90.8	0.51	0.08
44	At	PhAt-Cl					5.16	94.4	0.31	0.11
45	Cl	MesCl-Cl	18.1	81.4	0.58					
46	Br	MesBr-Cl	13	86.5	0.52					
47	I	MesI-Cl	8.82	90.7	0.43	0.07				
48	At	MesAt-Cl	5.65	94	0.26	0.11				
49	Cl	5fphcl-cl					15.2	84.1	0.69	
50	Br	5fphbr-cl					10.9	88.4	0.7	
51	I	5fphi-cl					5.87	93.4	0.6	0.1
52	At	5fphat-cl					3	96.5	0.34	0.14
53	Cl	Climidazole -Cl					14.8	84.4	0.8	
54	Br	Brimidazole -Cl					10.3	89	0.73	
55	I	Iimidazole -Cl					4.76	94.5	0.64	0.13
56	At	Atimidazole -Cl					2.31	97.1	0.39	0.19

QTAIM calculations

Table S8: Topology-based values at the X–Cl bond critical point (BCP).

All values given in atomic units (a.u.)

Code	G(r)	K(r)	V(r)	H(r)	Laplacian $\nabla^2\rho(r)$	ELF	ρ(r)	X-Cl Distance	X-CBP distance	Cl-CBP distance
9	1.25E-02	-1.67E-03	-1.08E-02	1.67E-03	5.66E-02	5.61E-02	1.64E-02	3.07E+00	1.466717	1.598901
10	1.38E-02	-1.33E-03	-1.25E-02	1.33E-03	6.06E-02	8.53E-02	2.00E-02	3.04E+00	1.488611	1.555691
11	1.83E-02	1.42E-03	-1.97E-02	-1.42E-03	6.75E-02	1.72E-01	3.01E-02	2.95E+00	1.476403	1.478122
12	1.98E-02	1.19E-03	-2.10E-02	-1.19E-03	7.45E-02	1.50E-01	3.00E-02	2.97E+00	1.49273	1.477197
17	1.16E-02	-1.68E-03	-9.90E-03	1.68E-03	5.30E-02	5.36E-02	1.55E-02			
18	1.31E-02	-1.39E-03	-1.17E-02	1.39E-03	5.81E-02	8.02E-02	1.90E-02	3.85E+00	1.818531	2.029324
19	1.68E-02	6.41E-04	-1.74E-02	-6.41E-04	6.47E-02	1.51E-01	2.73E-02	3.44E+00	1.680344	1.763625
20	1.88E-02	8.14E-04	-1.97E-02	-8.14E-04	7.21E-02	1.42E-01	2.85E-02	3.32E+00	1.666666	1.655634
41	2.35E-03	-7.13E-04	-1.63E-03	7.13E-04	1.22E-02	1.19E-02	3.74E-03	3.25E+00	1.632031	1.616661
42	6.06E-03	-1.41E-03	-4.65E-03	1.41E-03	2.99E-02	3.10E-02	8.85E-03			
43	9.33E-03	-1.34E-03	-7.99E-03	1.34E-03	4.27E-02	5.81E-02	1.39E-02			
44	1.14E-02	-1.27E-03	-1.01E-02	1.27E-03	5.06E-02	6.56E-02	1.63E-02			
45	3.13E-03	-9.17E-04	-2.21E-03	9.17E-04	1.62E-02	1.51E-02	4.78E-03			
46	5.28E-03	-1.29E-03	-3.98E-03	1.29E-03	2.63E-02	2.76E-02	7.86E-03			
47	8.46E-03	-1.35E-03	-7.11E-03	1.35E-03	3.92E-02	5.25E-02	1.27E-02			
48	1.06E-02	-1.31E-03	-9.25E-03	1.31E-03	4.75E-02	6.07E-02	1.52E-02			
53	1.09E-02	-1.82E-03	-9.13E-03	1.82E-03	5.11E-02	4.43E-02	1.41E-02	3.10E+00	1.473612	1.631032
54	1.21E-02	-1.79E-03	-1.03E-02	1.79E-03	5.56E-02	6.57E-02	1.70E-02	3.09E+00	1.496012	1.589794
55	2.01E-02	1.95E-03	-2.20E-02	-1.95E-03	7.26E-02	1.72E-01	3.18E-02	2.90E+00	1.43282	1.464409
56	2.26E-02	1.93E-03	-2.45E-02	-1.93E-03	8.27E-02	1.55E-01	3.29E-02	2.89E+00	1.436487	1.457949
57	6.13E-02	6.45E-02	-1.26E-01	-6.45E-02	-1.31E-02	7.80E-01	1.45E-01			
58	5.28E-02	4.13E-02	-9.41E-02	-4.13E-02	4.60E-02	5.68E-01	9.88E-02			
59	5.16E-02	4.16E-02	-9.31E-02	-4.16E-02	4.00E-02	5.85E-01	9.94E-02			
60	5.25E-02	3.04E-02	-8.29E-02	-3.04E-02	8.84E-02	4.83E-01	8.88E-02			

References

1. Engelage, E.; Schulz, N.; Heinen, F.; Huber, S. M.; Truhlar, D. G.; Cramer, C. J. *Chemistry* **2018**, *24* (60), 15983-15987.
2. Wang, Y.; Verma, P.; Jin, X.; Truhlar, D. G.; He, X. *Proc Natl Acad Sci U S A* **2018**, *115* (41), 10257-10262.
3. Kozuch, S.; Martin, J. M. *J Chem Theory Comput* **2013**, *9* (4), 1918-31.
4. Forni, A.; Pieraccini, S.; Franchini, D.; Sironi, M. *J Phys Chem A* **2016**, *120* (45), 9071-9080.
5. Anderson, L. N.; Aquino, F. W.; Raeber, A. E.; Chen, X.; Wong, B. M. *J Chem Theory Comput* **2018**, *14* (1), 180-190.
6. Sethio, D.; Raggi, G.; Lindh, R.; Erdelyi, M. *J Chem Theory Comput* **2020**, *16* (12), 7690-7701.
7. Weigend, F.; Ahlrichs, R. *Phys Chem Chem Phys* **2005**, *7* (18), 3297-305.
8. Siiskonen, A.; Priimagi, A. *J Mol Model* **2017**, *23* (2), 50.
9. Frisch, M. J.; Trucks, G. W.; Schlegel, H. B.; Scuseria, G. E.; Robb, M. A.; Cheeseman, J. R.; Scalmani, G.; Barone, V.; Mennucci, B.; Petersson, G. A.; Nakatsuji, H.; Caricato, M.; Li, X.; Hratchian, H. P.; Izmaylov, A. F.; Bloino, J.; Zheng, G.; Sonnenberg, J. L.; Hada, M.; Ehara, M.; Toyota, K.; Fukuda, R.; Hasegawa, J.; Ishida, M.; Nakajima, T.; Honda, Y.; Kitao, O.; Nakai, H.; Vreven, T.; Jr., J. A. M.; Peralta, J. E.; Ogliaro, F.; Bearpark, M.; Heyd, J. J.; Brothers, E.; Kudin, K. N.; Staroverov, V. N.; Keith, T.; Kobayashi, R.; Normand, J.; Raghavachari, K.; Rendell, A.; Burant, J. C.; Iyengar, S. S.; Tomasi, J.; Cossi, M.; Rega, N.; Millam, J. M.; Klene, M.; Knox, J. E.; Cross, J. B.; Bakken, V.; Adamo, C.; Jaramillo, J.; Gomperts, R.; Stratmann, R. E.; Yazyev, O.; Austin, A. J.; Cammi, R.; Pomelli, C.; Ochterski, J. W.; Martin, R. L.; Morokuma, K.; Zakrzewski, V. G.; Voth, G. A.; Salvador, P.; Dannenberg, J. J.; Dapprich, S.; Daniels, A. D.; Farkas, O.; Foresman, J. B.; Ortiz, J. V.; Cioslowski, J.; Fox, D. J. *Gaussian 09 Revision D.01*, Gaussian, Inc.: Wallingford, CT, 2016.
10. Lu, T.; Chen, F. *J Comput Chem* **2012**, *33* (5), 580-92.
11. *J Mol Graph Model* **2012**, *38*, 314-23.
12. Donald, K. J.; Pham, N.; Ravichandran, P. *The Journal of Physical Chemistry A* **2023**.
13. Glendening, E. D.; Badenhoop, J. K.; Reed, A. E.; Carpenter, J. E.; Bohmann, J. A.; Morales, C. M.; Karafiloglou, P.; Landis, C. R.; Weinhold, F. *NBO 7.0*, 7.0; Theoretical Chemistry Institute, University of Wisconsin Madison: Madison, Wisconsin, 2018.
14. Glendening, E. D.; Landis, C. R.; Weinhold, F. *J Comput Chem* **2019**, *40* (25), 2234-2241.
